# Cost of increasing access to artemisinin combination therapy: the Cambodian experience

**DOI:** 10.1186/1475-2875-7-84

**Published:** 2008-05-20

**Authors:** Shunmay Yeung, Wim Van Damme, Duong Socheat, Nicholas J White, Anne Mills

**Affiliations:** 1 Health Policy Unit, London School of Hygiene and Tropical Medicine, Keppel Street, London WC1E 7HT, UK; 2 Wellcome Trust – Mahidol University Oxford Tropical Medicine Research Programme, Faculty of Tropical Medicine, Mahidol University, 420/6 Rajivithi Road, Bangkok 10400, Thailand; 3 Department of Public Health, Institute of Tropical Medicine, Nationalestraat 155, B-2000 Antwerpen, Belgium; 4 The National Centre for Parasitology, Entomology and Malaria Control, 372 Monivong Boulevard, Phnom Penh, Cambodia

## Abstract

**Background:**

Malaria-endemic countries are switching antimalarial drug policy from cheap ineffective monotherapies to artemisinin combination therapies (ACTs) for the treatment of *Plasmodium falciparum *malaria and the global community are considering setting up a global subsidy to fund their purchase. However, in order to ensure that ACTs are correctly used and are accessible to the poor and remote communities who need them, specific interventions will be necessary and the additional costs need to be considered.

**Methods:**

This paper presents an incremental cost analysis of some of these interventions in Cambodia, the first country to change national antimalarial drug policy to an ACT of artesunate and mefloquine. These costs include the cost of rapid diagnostic tests (RDTs), the cost of blister-packaging the drugs locally and the costs of increasing access to diagnosis and treatment to remote communities through malaria outreach teams (MOTs) and Village Malaria Workers (VMW).

**Results:**

At optimum productive capacity, the cost of blister-packaging cost under $0.20 per package but in reality was significantly more than this because of the low rate of production. The annual fixed cost (exclusive of RDTs and drugs) per capita of the MOT and VMW schemes was $0.44 and $0.69 respectively. However because the VMW scheme achieved a higher rate of coverage than the MOT scheme, the cost per patient treated was substantially lower at $5.14 compared to $12.74 per falciparum malaria patient treated. The annual cost inclusive of the RDTs and drugs was $19.31 for the MOT scheme and $11.28 for the VMW scheme given similar RDT positivity rates of around 22% and good provider compliance to test results.

**Conclusion:**

In addition to the cost of ACTs themselves, substantial additional investments are required in order to ensure that they reach the targeted population via appropriate delivery systems and to ensure that they are used appropriately. In addition, differences in local conditions, in particular the prevalence of malaria and the pre-existing infrastructure, need to be considered in choosing appropriate diagnostic and delivery strategies.

## Background

Artemisinin combination therapies (ACTs) are now the officially recommended treatment for *Plasmodium falciparum *malaria in most malaria-endemic countries [1-3]. However, there are significant challenges in the actual implementation of this change in antimalarial drug policy. In the era of cheap monotherapies, namely chloroquine and sulphadoxine-pyrimethamine (SP), treatment for malaria was largely based on inaccurate clinical diagnosis and often obtained by patients from sources outside of the formal health sector. With ACTs currently costing around five to 10 times more than the traditional monotherapies, there needs to be a radical change in the approach to how they are funded and supplied in order to ensure that they are accessible and affordable to those who most need them. At a global level, support is gathering for the setting up of the Affordable Medicines Facilities -malaria, a global subsidy for ACTs, as recommended by the Institute of Medicine report "Saving lives, buying time" [4,5]. It is hoped that such a subsidy would enable ACTs to flow through the private sector as well as the public sector, to reach the poor and remote communities who are most vulnerable and, if subsidized heavily enough, displace monotherapies and sub-standard drugs. However, there is still significant uncertainty about how to best maximize access, whilst limit the wastage and risks associated with widespread inappropriate use of ACTs by those who do not have malaria [6,7]. Specific interventions to target the most biologically and economically at-risk include delivery mechanisms such as Village Malaria Workers (VMWs) and the wider use of rapid diagnostic tests (RDTs). Although the process and costs of policy change itself has been described [3,8], there is little experience of nationwide implementation and the cost of operationalizing such a policy change is largely unknown.

Cambodia was the first country to switch to a nation-wide policy of artemisinin combination therapy (ACT) using artesunate and mefloquine, for the first-line treatment of *P. falciparum *malaria in 2000 [9]. As in many tropical countries today, it was clear from the outset that there were a number of obstacles to successful implementation. These included the unavailability of any commercially available co-formulated or co-packaged ACTs; the low utilization of public health facilities in Cambodia; the high usage of antimalarials without biological confirmation of malaria; and the wide-spread availability of fake artesunate and mefloquine [10,11]. In order to address these problems, a number of innovative strategies were introduced including the local blister-packaging of artesunate and mefloquine in Phnom Penh, the deployment of HRP2-based rapid diagnostic tests (RDTs), and in certain areas, specific interventions to increase access to diagnosis and treatment through social marketing, Village Malaria Workers (VMWs) and malaria outreach teams (MOTs).

This study describes the incremental cost to the public sector of some of these interventions in order to inform the decision-making process in other countries embarking on the implementation of ACTs and feed into global advocacy on funding ACT scale-up [12].

## Interventions

### Blister-packaged artesunate and mefloquine

When the decision was made to recommend artesunate and mefloquine as the first-line treatment for uncomplicated *P. falciparum *malaria, there were no commercially available co-formulated or co-blistered products, and no local pharmaceutical companies with the capacity to produce blister-packages. However, it was felt that the blister packaging was essential to ensure that the drugs were correctly prescribed by health providers, and correctly taken by patients. In addition, in view of the prevalence of fake drugs, it was felt that pre-packaging drugs would enable both patients and health workers to be assured of drug quality, as only drugs with internationally recognized Good Manufacturing Practice (GMP) would be used. The decision was therefore made to blister package artesunate and mefloquine locally as a temporary measure. This was done by the National Malaria Centre (CNM) with support from World Health Organization (WHO). A room in the Central Medical Stores building was renovated, new packaging and printing machines were installed and staff given appropriate training. Maximum production was estimated at around 2,000 tablets/day or 40,000 blisters per month. In reality only 56,794 packages were officially produced in 2001[13].

The recommended regime was artesunate given over three days (4 mg/kg/day) and mefloquine to be given as a split dose on the first day (Table 1). The tablets were pre-packaged in three different packages based on weight and age as shown in Table 1. Children under six years of age were recommended to receive five days of daily rectal artesunate (but this is now being replaced with oral artesunate and mefloquine). Packages of artesunate and mefloquine were packaged for the public sector in plain boxes and labelled with "A+M" and a warning of "not for sale". The artesunate and mefloquine regimes for the two older age groups were also made available in the private sector in social marketing scheme that was initially piloted in two districts, and then launched nationwide in March 2003. The drugs were packaged in more attractive boxes and sold to the private sector as "Malarine^®^" at a subsidized price The packaging process has since been contracted out to the Cambodian Pharmaceutical Enterprise (CPE), a partly government owned pharmaceutical company, and the social marketing of Malarine^® ^has been contracted to Population Services International (PSI).

**Table 1:** Original dosage of artesunate (A) in 50 mg tablets and mefloquine (M) in 250 mg tablets*

**Packages of artesunate and mefloquine**	**Weight**	**Age**	**Day D**_1_		**D**_2_	**D**_3_
					
			**Morning**	**Evening**		
A+M2	16 – 24 kg	6 – 10 years	1M+1A	1M+1A	2A	2A
A+M3	25 – 34 kg	11 – 14 years	1M+1A	2M+2A	3A	3A
A+M4	35+ kg	15+ years	2M+2A	2M+2A	4A	4A

* The dosages are being modified in 2008

### Rapid diagnostic tests (RDTs)

Malaria transmission in Cambodia is generally low and seasonal so a positive blood smear or positive RDT has a high predictive value for infection with *Plasmodium falciparum*. One of the keystones of the new policy was the provision of accurate biological diagnosis using RDTs before treatment, to replace the inaccuracies of clinical diagnosis. As around 90% of malaria in Cambodia was due to *P. falciparum*, Paracheck^® ^(Orchid Biomedical Systems), a *P. falciparum *– specific PfHRP2-based RDT was introduced for use both in health centres without microscopes and in the private sector alongside Malarine^®^. The RDT was also used by trained village malaria workers (VMWs) and malaria outreach teams (MOTs) as described later.

### Interventions to improve access to early diagnosis and appropriate treatment

In order to improve access to diagnosis and treatment, a number of specific interventions were developed independently by different organizations. This study describes the cost of two of these interventions aimed at increasing access to free or affordable diagnosis and treatment in the community: Malaria Outreach Teams and Village Malaria Workers.

#### Malaria Outreach Teams

Malaria outreach activities in Anlong Veng District, Oddor Meanchey province were set-up, run and funded by Médecins Sans Frontières (MSF) as part of their programme of support in that area. This remote and heavily forested area had remained a Khmer Rouge (KR) stronghold until 1999 and therefore lacked government-supported health facilities. The collapse of KR power resulted in an influx of non-immune migrants from all over Cambodia, who came in search of farmland and to collect forest products.

One of the major health problems as new settlers moved into these areas were outbreaks of malaria. Between May and August of 1999, in the health centre alone, there were over 2000 confirmed malaria cases, 400 hospitalizations and 18 deaths with malaria accounting for one third of all outpatient and two thirds of all inpatient cases [14]. At the time, the national antimalarial guidelines for uncomplicated *P. falciparum *malaria in that area was a single dose of mefloquine (20 mg base/kg), and for complicated malaria, quinine and tetracycline. In response to the outbreak and the known problem of drug resistance, MSF switched first-line treatment to an ACT of artesunate and mefloquine (A+M) and set up malaria outreach teams (MOTs) consisting of teams of two people who went out daily from the health centre to the settlements, in order to diagnose and treat malaria using RDTs and A+M. There were initially two teams, expanding to four teams with the aim of visiting each settlement once or twice per week depending on population movement, road conditions and information about suspected malaria outbreaks. The population of the district in 2001 was estimated to be 19,029 in 64 settlements (Goubert L, personal communication).

### Village Malaria Workers (VMWs)

The first experience of using Village Malaria Workers in Cambodia came from a community based trial for insecticide treated bed nets (ITNs) in 30 villages in Rattanakiri in the Northeast of the country in 2001. This is a remote, heavily forested area, sparsely populated by ethnic minorities, with low access to any kind of health service. Malaria transmission in Rattanakiri is relatively high with cross-sectional parasite prevalence rates of between five and 57% [15]. In order to address ethical concerns about having a control group without any interventions, Village Malaria Workers (VMW) were introduced in all villages. The VMWs were trained to perform RDTs on any villagers suspected of having malaria and to provide treatment as per the national guidelines. They were supervised and re-supplied monthly by the provincial malaria staff with support from the National Malaria Control Programme. The resulting data from this passive surveillance system exposed the scale of the malaria problem and demonstrated that VMWs provided a practical means of access to biological diagnosis and appropriate treatment. A further pilot project was undertaken in 10 ethnic Khmer villages in Koh Kong province in the South [16], and the scheme has now been scaled up to cover 300 villages in 10 provinces with funds from the Global Fund for AIDS, tuberculosis and malaria (GFATM) [17,18].

### Costing

Costing was performed from the perspective of the provider. The incremental costs of the interventions are described in order to aid generalization to other settings. Costing was facilitated by the fact that the interventions were specific to the change in policy to ACTs, and implemented with new funds obtained for this purpose. Costs were obtained from receipts, expenditure accounts, budgets, logbooks and reports. Where these were not available, estimates were obtained from WHO, MSF and CNM staff who also provided estimates on time allocated to specific interventions. Costing of the VMW intervention was based on the Global Fund budget. The useful life of capital goods was assumed to be six years for cars and five years for all other goods, with costs annualized at a rate of 3%. All costs were converted into US$ at an exchange rate of US$1 = 3,900 Cambodian riel (which has remained stable over the last seven to eight years).

The cost of blister packaging included the capital costs of starting up the packaging facility (renovations, machinery, consultancies and training), fixed recurrent costs (overheads, maintenance and salaries) and those that varied by production volume (packaging materials and drugs). Actual expenditure on salaries for machine operators was not available and average monthly earnings in the manufacturing industry in Cambodia were used.

The cost of RDTS included the cost of storage and wastage due to spoilt and repeat test. In addition the cost of training and supervision costs were also estimated.

For the delivery interventions, the costs included the diagnostic tests and the blister-packaged drugs and the fixed costs of each approach. The latter included the "basic" fixed costs of training (including training materials, transport and per diems for trainers and participants), overheads, transport and salaries. In addition the project start-up costs and the cost of project co-ordinators were estimated separately in order to facilitate comparison with other settings. For each intervention, the level of activity in terms of number of suspected malaria patients tested and number of patients with falciparum malaria treated was obtained in order to estimate the cost per case seen and treated for each intervention. Both VMWs and MOTs were closely supervised with cross-checking of RDT results with drugs stocks ensuring that compliance to test results was acceptable and that the practice of providing ACTs to test-negative patients was minimized.

## Results

**Figure 1:**
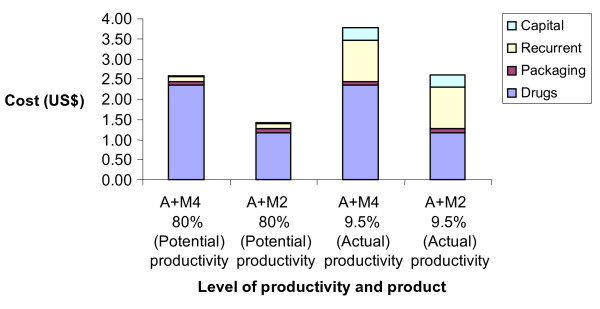
Breakdown in costs of blister-packaged drugs.

The cost of the packaging materials and drugs is shown in Table 2 and the total costs of the end-products are shown in Table 3. The total costs of the blister-packaged drugs were between $2.60 for the package for the A+M2 and $3.77 for A+M4. The cost of Malarine^® ^was slightly more than A+M4 because of the higher cost of the artesunate used (different dosage artesunate tablets were used, partly in order to differentiate between the two types of products). The cost of packaging material was $0.09 per package for all packages. At the actual rate of production, the salary cost was $0.34 per package and overhead costs $0.71 per package, resulting in packaging cost alone of $1.13 per package. If production were to increase to full capacity, then the total cost of packaging would be reduced more than five-fold to $0.19 per package, resulting in the total costs of A+M2 and A+M4 being considerably lower, at $1.42 and $2.59 respectively. Figure 1 shows that when the costs of the product are broken down, at the current level of productivity, the drug costs accounted for only 45% and 62% of the cost of A+M4 and A+M2 respectively, compared to 83% and 90% at the desirable level of productivity.

**Table 2:** Cost of packaging and tablets

**Cost of packaging**^1^	**Cost per blister (US$)**
Aluminium foil	0.0267
PVC	0.0115
Individual boxes	0.0340
Large boxes (for 100 individual boxes)	0.0048
Ink for printing on box^2^	0.0020
Package insert	0.0010

**Cost of drugs**	**Cost per tablet (US$)**^3^

Mefloquine 250 mg (Mepha^®^)	0.3696
Artesunate 50 mg (Gui Lin^®^)	0.0725
Artesunate 200 mg (Mepha ^®^)	0.3323

^1^The cost of the aluminium foil and PVC included freight and storage. Other products were available locally and the cost is the price paid inclusive of delivery and storage.

^2^Although the printing costs for the Malarine^® ^products was higher than A+M because of the amount of coloured ink used, only an average cost of ink was available.

^3^Including 5% wastage and 3% freight.

**Table 3:** Cost of blister-packaged artesunate and mefloquine

**Drug package**	**Actual cost (at 9.5% productivity)**		**Potential cost (at full productivity)**	
	
	Recurrent costs	Recurrent and capital costs	Recurrent costs	Recurrent and capital costs
A+M2 (Art 50 mg × 6 + Mef 250 mg × 2)	2.307	2.595	1.386	1.420
A+M3* (Art 50 mg × 9 + Mef 250 mg × 3)	2.894	3.182	1.973	2.007
A+M4 (Art 50 mg × 12 + Mef 250 mg × 4)	3.480	3.769	2.560	2.594
Malarine^® ^(adult) (Art 200 mg × 3 + Mef 250 mg × 4)	3.608	3.896	2.688	2.722

*Same cost for Malarine junior ^®^

The basic cost of RDTs, inclusive of freight, storage and wastage, was $0.83 for Paracheck^®^. Training was estimated to cost $40.50 per health centre staff trained (based on a one-day training course).

Tables 4 and 5 summarize the results of the cost analysis for the MSF malaria outreach project and the VMW scheme. It can be seen that excluding the cost of start-up and co-ordinator, the annual fixed cost per capita of the outreach scheme, at $0.44, was less than the VMW scheme at $0.69. However, because a much smaller proportion of the population was seen and treated by an outreach worker than a VMW, the annual fixed costs per malaria case treated for the latter was $5.14 compared to $12.74 in the malaria outreach scheme. Including start up costs (annualized over five years), and the annual salary of co-ordinators increased these estimates significantly, to $7.48 for the VMW intervention and $21.12 for the malaria outreach intervention.

**Table 4:** Summary of the annual fixed costs of the outreach and VMW interventions (not including RDTs and drugs)

	**Cost (US$)**	
	
	**Outreach**	**VMW**
**Basic annual fixed running costs**		
**(excluding expatriate co-ordinator and start-up)**		
Personnel (including cost of supervision)	5,422	20,270
Transport	2,224	37,850
Other (equipment, overheads)	739	10,738
**Sub-total**	**8,385**	**68,858**
**Additional costs**		
Annual salary of co-ordinator (20% allocation for outreach, 100% for VMW)	4,643	24,000
Initial start-up cost annualized over 5 years (Total $4,710 for outreach and $40,000 for VMW)	869	7,384
**Sub-total**	**5,512**	**31,384**
**Total annual cost**	**13,897**	**100,242**

**Table 5:** Estimated activity and cost of interventions per capita, per patient seen and per patient treated

	**Outreach**	VMW*
Population	19,029	100,000
Number seen and tested	3,152	57,360
Number *P. falciparum *cases treated	658	13,407
		
Basic annual fixed cost**		
-per capita	$0.44	$0.69
-per patient seen and tested	$2.66	$1.20
-per PF patient treated	$12.74	$5.14
		
**Total annual cost (including test and drugs)**		
-per capita	$0.67	$1.51
-per patient seen and tested	$4.03	$2.64
-per PF patient treated	$19.31	$11.28

*Based on estimates from pilot project and the Global Fund budget

**Without start-up and co-ordinator costs

Based on the activity data obtained from the outreach programme and from the VMW pilot study, the total cost inclusive of RDT and drug costs, was estimated. Assuming that RDTs cost $0.83 per test and A+M cost $2.594 per course, the total annual cost of the programmes was $19.31 per capita for the outreach intervention and $11.28 per capita for the VMW interventions (Table 5). If drug costs were reduced to $1 per dose, the predicted cost of dihydroartemisinin-piperaquine, then the total annual costs would be approximately $17.72 and $9.69 per capita respectively.

## Discussion

In this paper, the costs of interventions associated with the roll-out of ACTs in Cambodia has been described in order to assist countries which are now in the process of implementing a change in policy to ACTs.

At the time when Cambodia change policy to artesunate and mefloquine, there was not other choice than to co-blister the drugs in a packaging facility developed specifically for that purpose. This study shows that at the inefficiencies of packaging at this scale were considerable. The low productivity was due to a number of factors including bottle-necks during the drug procurement process and the inefficiencies related to small demand-based production. Production has now been moved to Cambodian Pharmaceutical Enterprise, where staff can be moved to other production lines when not working on A+M resulting in greater efficiency. However, the local blister-packaging of artesunate and mefloquine has not been straightforward and much technical support has been required in order to ensure that the packaging process is of acceptable quality. This has resulted in critical delays and shortages in supply and there is a consensus that a switch should be made a soon as possible to a commercially packaged good-quality co-formulated ACT.

Several commercially produced co-formulated ACTs are now available for less than $2 including artemether-lumefantrine (Co-artem^®^) and dihydroartemisinin-piperaquine (Duo-Cotexcin^®^). Unfortunately artemether-lumefantrine, which is the only co-formulated drug on WHO's pre-qualification list, is relatively ineffective in Cambodia [19]. On the other hand dihydroartemisinin-piperaquine has been shown to be highly efficacious in Cambodia and is a possible replacement for A+M for the treatment of uncomplicated falciparum malaria [20]. Co-formulated artesunate and mefloquine is unlikely to be deployed because of a preference of switching to a new combination with less side effects and lower levels of resistance to the partner drug. For now, therefore, the recommended treatment in Cambodia continues to be blister-packaged artesunate and mefloquine.

From the cost analysis of delivery interventions, it is evident that interventions to increase access to treatment, can incur substantial costs especially when start-up costs and co-ordinator salaries are included. This is particularly the case where programmes are malaria-specific and the incidence of malaria is not that high, especially if staff are not re-allocated to work on non-malarial problems in months of low malaria transmission. The incremental costs described in this analysis are probably particularly high because of the limited infrastructure in the districts where these interventions took place and because malaria control management was still centralized. In neither case were there pre-existing services for these remote communities. In Anlong Veng, the outreach programme was based out of the MSF-supported health centre which provided office space and laboratory support for the malaria outreach workers. For the VMW programme, the supervision and re-supply of the VMWs was done both by the provincial malaria supervisor and by staff from the CNM.

In comparing the two interventions, the fixed cost per case tested and per case treated in the VMW scheme was almost half the cost in the outreach scheme. This is because the prevalence of malaria was higher in the area covered by the VMW scheme in Rattanakiri from where the estimates for the projected number of visits and cases came. The VMWs were also more convenient and accessible than outreach and were more often consulted. Recent data from the scaled-up VMW intervention confirmed high rates of consultation and high rates of parasite positivity in symptomatic cases which would result in even lower fixed costs per patient tested and treated [17]. In addition, there are plans to broaden the remit of the VMWs so that they can also treat diarrhoeal disease and acute respiratory illness in children. Not only will this provide a more comprehensive service for the remote communities, but it will also substantially improve the cost-effectiveness of the intervention.

However, as explained, the populations covered by these two interventions were quite different and it was felt that VMWs would not have operated successfully in Anlong Veng district because of the disparate and transient nature of population. It cannot, therefore, be assumed that VMWs are the more cost-effective option in all settings. Nonetheless, the cost of the outreach programme, at $12.74 per positive case, appeared to be considerably more than that estimated by Ettling *et al *for a similar intervention in Mae Sot, Thailand, where diagnosis was by microscopy and treatment was with a single dose of mefloquine [21]. Despite the slide positivity rate in Thailand being only 5%, the average provider cost per positive case was equivalent to $2.57 and $0.13 per smear ($US1 (2005) = 35 baht (1990). It is not clear why there is such large difference between the estimated costs of the two programmes.

There have been a number of other descriptive studies of Village Malaria Workers, however in most, costing data were not available. One cost-minimization analysis was performed on a small community based intervention in Brazil. Volunteer bar owners in a remote mining town were trained to use the ParaSight-F^® ^test (a similar test to Paracheck^®^) to diagnose malaria and to treat positive cases with mefloquine monotherapy. The study compared the direct costs (patient and provider) incurred with the intervention compared to the costs prior to the intervention, when the main source of treatment was the government clinic and hospital located 32 kilometres away. It was found that net savings were $60,900 or $81 per person per year [22,23].

It is important to recognize that the interventions described here can both complement and substitute for facility-based approaches. The choice and balance of different approaches needs to be tailored according to the availability of resources and local conditions, and adapted as the situation changes. In particular, malaria prevalence will affect the relative efficiency of different strategies [21,24]. In both the VMW scheme and the outreach scheme, the nearest health centres provided co-ordination, support and supervision and were also essential in being a place where patients could be referred to. It was therefore important to ensure that these were functioning well and that resources were allocated appropriately to enable this to be the case.

There are few published data that include both the costs and the effect of these interventions from the community perspective. In a related paper, antimalarial drug usage behaviour and household costs in areas with and without VMWs and outreach clinics are compared in order to contribute to the available literature [25].

## Conclusion

As malaria-endemic countries start to roll-out ACTs and the global community consider the setting up of a global subsidy, it is important to consider the non-drug costs of roll-out. If this includes the cost of packaging drugs locally, then it is important to consider the efficiencies of scale that result from producing larger volumes of packaged drugs. Of even more importance is to consider the need to access vulnerable communities and the substantial additional investments required in order to reach them via appropriate delivery systems such as village volunteers. In addition, differences in local conditions, in particular the prevalence of malaria and the pre-existing infrastructure, should be considered in choosing appropriate diagnostic and delivery strategies. The costs of starting-up projects and the expertise required for supervision may be considerable and must be factored in when budgeting for scaling-up.

## Authors' contributions

SY designed the study, carried out the data collection and analysis and drafted the paper. WVD facilitated the collection of data and made substantial contributions to the writing of the paper. DS participated in the study design and co-ordination of fieldwork. NJW conceived of the study and participated in writing the paper. AM participated in the study design, data interpretation and helped to draft the paper. All authors read and approved the final manuscript.
